# Changes in Ankle Range of Motion, Gait Function and Standing Balance in Children with Bilateral Spastic Cerebral Palsy after Ankle Mobilization by Manual Therapy

**DOI:** 10.3390/children7090142

**Published:** 2020-09-18

**Authors:** Pong Sub Youn, Kyun Hee Cho, Shin Jun Park

**Affiliations:** 1 Department of Physical Therapy, Kyungbok University, Namyangju-si 425, Korea; psyoun@kbu.ac.kr; 2 AVENS Hospital, Dongan-gu, Anyang-si 307, Korea; 201876402@yiu.ac.kr; 3 Department of Physical Therapy, Gangdong University, Daehak-gil 278, Korea

**Keywords:** ankle joint, cerebral palsy, COP, gait, join mobilization

## Abstract

The aim of this study was to investigate the effect of ankle joint mobilization in children with cerebral palsy (CP) to ankle range of motion (ROM), gait, and standing balance. We recruited 32 children (spastic diplegia) diagnosed with CP and categorized them in two groups: the ankle joint mobilization (*n* = 16) group and sham joint mobilization (*n* = 16) group. Thus, following a six-week ankle joint mobilization, we examined measures such as passive ROM in ankle dorsiflexion in the sitting and supine position, center of pressure (COP) displacements (sway length, area) with eyes open (EO) and closed (EC), and a gait function test (timed up and go test (TUG) and 10-m walk test). The dorsiflexion ROM, TUG, and 10-m walk test significantly increased in the mobilization group compared to the control group. Ankle joint mobilization can be regarded as a promising method to increase dorsiflexion and improve gait in CP-suffering children.

## 1. Introduction

Cerebral palsy (CP) is a non-progressive upper motor neuron lesion where common motor disability occurs. Other manifestations of CP, such as loss of posture control and lack of movement, may also result in the development of musculoskeletal problem [1,2]. These problems arise because of spasticity and occurs in the hip, knee, and ankle joints [3]. Spasticity is a form of velocity-dependent resistance, or a motor disorder [4]. Children with spastic diplegic CP had greater spasticity at the ankles (more distal part) compared with the knees [5]. The ankle spasticity is related to the limited ankle joint movement [6]. In particular, the limited ankle joint movement in children with CP are closely related to gait and balance performance [7,8]. Therefore, the improvement of balance and gait ability regarding ankle function is one of the goals in rehabilitation for the musculoskeletal problems of children with CP [9,10].

Children with CP are observed to have a limited ankle ROM than typically developing children [11]. The limited ankle ROM of children with CP is also associated with higher tissue stiffness, increased reflexive torque of the gastrocnemius and soleus [11], and spasticity and weakness of the ankle joint [12]. The limited dorsiflexion ROM causes changes in contractile tissue as well as non-contractile tissue and is a common problem for children with CP [13,14].

A limited ankle ROM during gait and balance performance is an identified problem in children with CP [8,15,16]. On the other hand, an ankle ROM improvement may facilitate gait and balance performance [17,18]. For the optimal state of ankle dorsiflexion, it is essential to have a better understanding of the mechanism of limited ankle ROM, and to increase ankle ROM, not only osteokinematics but also arthrokinematics are required. Osteokinematics are simply movements of bones at the joints (flexion/extension, abduction/adduction, and internal rotation/external rotation), and arthrokinematics are small movements of bones at the joint surface (rolls, glides/slides, and spins).

Manual mobilization serves as an important component of neurorehabilitation to treat spasticity and limited ankle movements [19,20,21,22]. Manual mobilization is a passive movement performed to relieve pain, complete joint motion, and restore arthrokinematics rather than osteokinematics. Ankle joint mobilization can improve the associated limited ankle ROM characteristic of this condition, especially dorsiflexion [23,24]. Increased dorsi flexion ROM through joint mobilization improved the ankle kinematic changes during walking and [18] and static postural control [25]. These results seem to have a favorable effect on the sensorimotor function and arthrokinematic motion of the ankle [24,25].

Joint mobilization provides a well-suited intervention for ankle rehabilitation, but the effectiveness of a randomized control trial study in children with CP has not been confirmed. As part of manual therapy, there are spine manipulation studies for cerebral palsy [26,27]. Manipulation is a high-velocity (thrust) technique, while joint mobilization is a relatively safe technique with low-velocity techniques [28]. Manual therapy has been supported by several researchers that it can be used as a separate intervention method to treat secondary problems of musculoskeletal system in patients with spasticity (CP or stroke) [19,20,21,26,27,29,30,31,32,33].

Therefore, the purpose of this study is to investigate the effect of ankle joint mobilization to improve ankle ROM, standing balance, and gait in children with CP.

## 2. Materials and Methods

### 2.1. Study Design

This study was single blind, randomized controlled trial with two groups: a mobilization group (ankle joint mobilization) and control group (sham joint mobilization); individuals were randomly assigned using sequentially numbered, opaque-sealed envelopes. The base-line test was performed after obtaining a written informed consent from participants and their legal representatives. The base-line test is ankle dorsiflexion ROM, COP displacements, and gait function (timed up and go test, 10-m walk test). After the baseline-test, children with CP were randomly divided into 2 groups: the mobilization group (ankle joint mobilization, *n* = 16) and control group (sham joint mobilization, *n* = 16). The allocation ratio of the mobilization group/control group was 1:1. The gross motor skills of children with CP can be categorized into 5 different levels using the gross motor function classification system (GMFCS). Since the GMFCS level can provide a confounding interpretation of the results, this study used a stratified block randomization form of the level of GMFCS (1 or 2) [34]. Children with CP were blinded to their treatment. The examiner was blinded to the group allocation. Only the physiotherapist who performed the joint mobilization knew the group to which the participants belonged to. The end-line test was performed after 6 weeks. This study was performed in accordance with the Declaration of Helsinki and research work was approved by the Institutional Review Board of Yong-in University (2-1040966-AB-N-01-20-1812-HSR-127-10).

### 2.2. Participants

**Figure 1:**
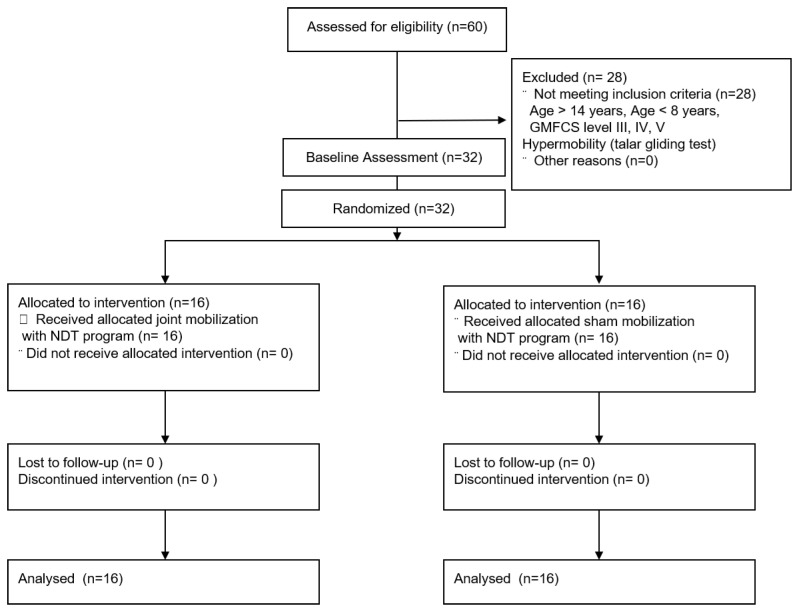
The flowchart of recruitment.

A total of 32 children with CP were recruited from the Bundang Jesaeng general hospital in Gyeonggi do, Korea, February to May 2019. The inclusion criteria were (1) school-aged cerebral palsy children (8 to 14 years) (Mehraban et al. 2016); (2) a diagnosis of spastic diplegia; (3) gross motor function classification system (GMFCS) level I or II [35]; (4) hypomobility according to a 5-point posterior talar gliding test [36]; (5) ability to walk 10 m or more independently; and (6) children with CP to follow verbal directions. Exclusion criteria were (1) a history of selective dorsal rhizotomy and lower extremity orthopedic surgery; (2) botulinum toxin injections in leg muscles during the preceding year; and (3) visual disorder. The detailed study plan is depicted in Figure 1.

### 2.3. Sample Size Calculation

G*Power 3.19 (Heinrich Heine University, Dusseldorf, Germany) was used for sample size calculation. The sample size of this study was calculated based on the pilot test. Eight children with CP (four children in the joint mobilization group and four in the sham mobilization group) were involved to calculate the subjects needed for this study. The effect sizes of 1.30 (left) and 1.40 (right) were derived using the mean and standard deviation of dorsi flexion ROM in the supine position among the main outcomes. Based on the effect size of 1.30, input of a confidence level of 95%, and power of 80%, the total required sample size was 22. In this study, 32 participants were selected considering dropout. The initial participants were not included in the sample of 32.

### 2.4. Intervention Methods

The period of the ankle intervention in this study was 6 weeks. All participants received ankle intervention 30 times (5 sessions per week) over a 6-week period. The mobilization and control groups received the neurodevelopment treatment (NDT) program for 6 successive weeks. The NDT program was performed to improve trunk control. The NDT program consisted of 20 min of trunk muscle exercise and upper extremity exercise (a reaching task of the upper limb for mobility, and trunk control for stability in the sagittal, coronal and transverse planes). The principles of NDT are trunk control in the sitting and standing positions [37]. The mobilization group additionally received (12 min) ankle joint mobilization. The control group additionally received (12 min) sham mobilization.

### 2.5. Ankle Joint Mobilization

**Figure 2:**
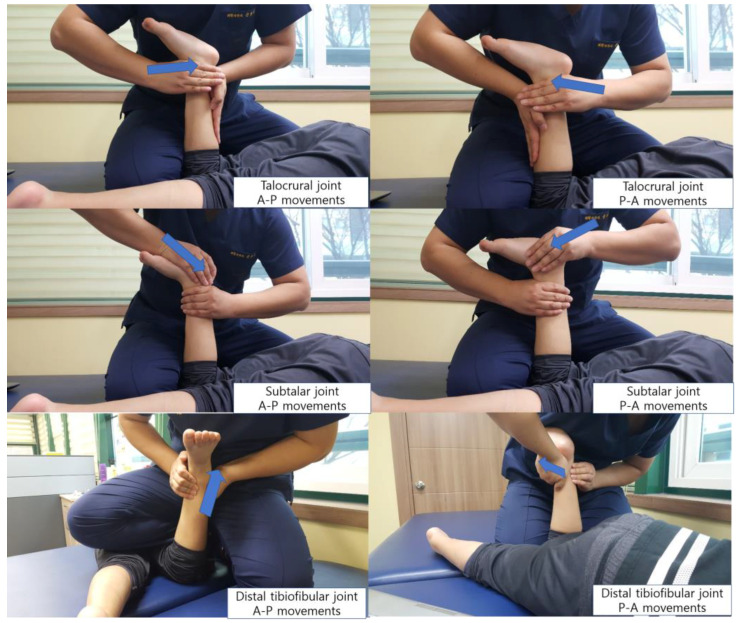
The ankle joint mobilization.

Ankle joint mobilization was performed to improve dorsiflexion ROM. Joint mobilization was performed by one physiotherapist certified in IMTA Maitland concept level 1, with over 10 years of neurodevelopmental treatments experience. After manual evaluation, ankle joint mobilization was carried out in the distal tibiofibular joint, talocrural joint, and subtalar joint. The manual evaluation for ankle joint mobilization procedure is depicted in Figure 2. Manual evaluation to determine the direction of the joint mobilization is as follows. The joint mobilization group recorded the direction of hypomobility among the hypermobility, normal and hypomobility, and performed large-amplitude, rhythmic oscillations (grade III) in the direction of the hypomobility [38]. In this study, except for the distal tibiofibular joint, joint mobilization was applied in the AP direction, and participants received about 50 oscillations per set with 1 min of rest between sets. The rest time was 1 min and applied to both legs for a total of 12 min by applying joint mobilization to the opposite leg during a rest time of 1 min. Therefore, joint mobilization was applied to each of the three ankle joints for 4 min, and the oscillations technique was applied in 2 sets per one leg.

### 2.6. Sham Mobilization

The sham mobilization visually resembles the joint mobilization. The sham mobilization seems to perform the same action as ankle joint mobilization, but only manual contact is performed because there is no direction of oscillations.

### 2.7. Assessments

The main outcome measure was ROM in ankle dorsiflexion. The secondary outcome measure was COP displacements and the gait function test. All measurements were performed on the barefoot.

### 2.8. Ankle Dorsiflexion ROM

Ankle dorsiflexion ROM was measured in the supine position and sitting position [19,39]. For the measurement, a goniometer (goniometer, jamar) was used. To measure the supine position, the participants were in the supine position on a treatment table in a knee extension posture, a sitting position. Measuring of the participants was in the sitting position and with their hip and knees’ flexion in 90°. The goniometer axis is located on the lateral malleolus and the stationary arm is located parallel to the fibular head. The movement arm was then the lateral aspect of the fifth metatarsal bone. The examiner fixed the tibial bone and pushed the foot of the participant toward the dorsiflexion. The dorsiflexion ROM was measured where the end-feel was felt, and no further movement occurred. Measurement of ankle joint ROM using a goniometer has a high reliability [40].

### 2.9. COP Displacements

**Figure 3:**
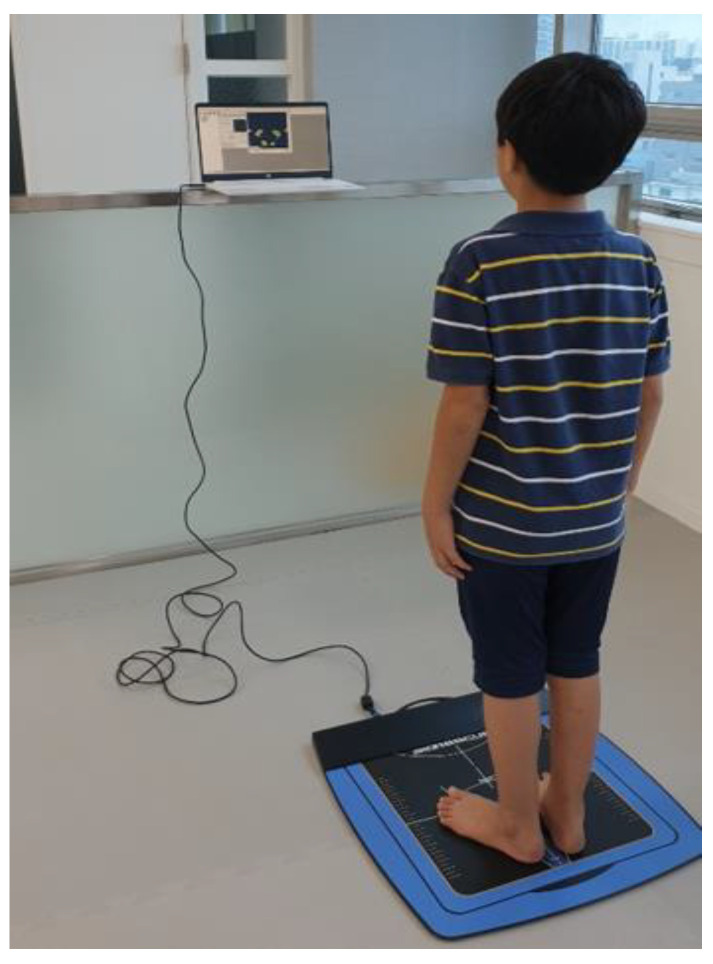
The center of pressure (COP) displacements.

COP displacements was measured in a quiet standing position. The COP displacements evaluation is depicted in Figure 3. For the measurement, an AP1153 BioResque (RM Ingenierie, Rodez, France) was used. BioResque is a pressure force platform with 1600 sensors embedded. Participation was achieved by aligning the individuals’ bare feet at the 30° leader line indicated above the measurement field of 400 × 400 mm, and holding the standing position for 30 s. At this time, the static sway length (cm) and static sway area (mm^2^) of the COP displacement value were measured. COP displacements were measured for an eyes closed and opened condition. The smaller the measured value, the better the standing balance ability.

### 2.10. Gait Function

The timed up and go test was used to assess mobility and balance. The starting position was sitting on a chair without armrests with the hip, knees, and ankle bent in a 90° angle. Participants had to get up from a chair, walk 3 m, return, and sit back in the chair. Measurements were taken after the “start” verbal cue provided by the examiner and recorded until the hips touched the chair. When measuring, the participants were barefoot. The TUG test is suitable for reliable and responsive for measuring functional mobility and dynamic balance of children with CP in GMFCS levels I–III [41]. A 10-m walk test was used to assess gait speed. In this study, a 14-m walkway was used. A stopwatch was used for the measurement, with the starting position in the standing position. When the participant started walking, the time required to walk the 10-m walking distance was measured, excluding the initial point 2 m (acceleration section) and the last point 2 m (deceleration section). The 10-m walk test provides high reliability in children with CP [42,43].

### 2.11. Statistical Analysis

Data analysis was done using SPSS version 20 software (IBM Corp, Armonk, NY, USA). The confirmation of homogeneity and a normal distribution was verified by means of the K-S tests and independent *t*-tests and chi-square tests. The effects of intervention on dorsiflexion ROM, COP displacements, TUG, and 10-m walk test were examined with a two-way repeated-measures analysis of variance (two-way RM ANOVA). The difference between the initial test and post-hoc test was within-group (time). The mobilization group and control group were between-group (group by time or interaction). If a significant difference appeared in the main effect or interaction, the within-group difference was measured with a paired *t*-test, whereas a between-group difference was calculated using an independent *t*-test. The alpha of statistical significance was set at 0.05.

## 3. Result

The general characteristics confirmed the homogeneity between the two groups (Table 1).

### 3.1. Change in ROM Dorsiflexion

The experimental groups showed a significant increase in ankle ROM in the sitting and standing position. In addition, the experimental group displayed a significant increase in all ankle ROM compared to the control group (Table 2).

### 3.2. Change in COP Displacements, TUG, and 10-m Walk Test

Both the experimental and the control groups displayed a significant decrease in the static sway length and area in the eyes opened condition, eyes closed condition, TUG, and 10-m walk (Table 3). In addition, the experimental group had a significantly increased TUG and 10-m walk test than the control group. However, there was no significant difference between the two groups. However, there was no significant difference between the experimental group and control group in COP displacements (Table 3).

## 4. Discussion

This study found that ankle joint mobilization to improve ankle movements increased ankle ROM and gait function. Moreover, this study demonstrated that ankle joint mobilization is more effective for ankle ROM and gait than sham mobilization. This indicates the importance of ankle joint mobilization in orthopedic management for ankle rehabilitation in children with CP. The strength of this study is that it is the first time in children with CP that joint mobilization was applied to ankle joints in a manual therapy technique. A direct comparison is difficult, but it is consistent with other studies showing that additional joint mobilization is more effective in increasing ankle ROM and gait than conventional rehabilitation [19,24].

When compared with studies regarding the change in passive stretching exercises applied to calf muscles on dorsiflexion ROM [18,44], this study confirmed the positive change effects of passive joint mobilization, such as improved dorsiflexion ROM, TUS, and 10-m walk test. Children with CP exhibit spasticity in calf muscles, so passive stretching has been applied to reduce the spasticity, relaxation, and elongation effect on muscles in previous studies [18,44,45].

However, spasticity exacerbates joint contracture and muscles weakness, as well as changes in the muscle contractile properties [46]. Ankle joint mobilization can be applied to reduce the spasticity of the soleus muscles and [33] restore ankle joint flexibility [19,32]. In addition, ankle joint mobilization causes articular reflexogenic effects, increasing dorsiflexor muscle strength [47]. It has been found that for stroke patients, joint mobilization is a way to increase a variety of ankle ranges of motion rather than stretching exercises [19]. Therefore, joint mobilization can be used as an intervention method to increase ankle mobility in children with CP.

The ankle and knee ROM of children with CP is highly correlated with the energy expenditure index, which means gait efficiency [15]. Ankle joint mobilization increased the ankle dorsiflexion ROM and speed of the sit-to-stand performance [32]. Among the variables measured in this study, the timed up and go test included sit-to-stand. The increased ankle ROM can increase the speed of the sit-to-stand performance, so the timed up and go test may be improved. In addition, ankle joint mobilization with movement can improve gait speed [21]. The posterior talar glide can be increased through joint mobilization [24,48]. Increased posterior talar glide increases dorsiflexion before heel-off and time to heel-off during gait movements [24]. Improving gait velocity through ankle joint mobilization can be considered to affect the dorsiflexion increase during gait movements [19,21,24]. Consequently, ankle joint mobilization improved the gait speed by increasing dorsiflexion during gait movement.

Another finding of this study was that there was no change in the standing balance measurements after additional joint mobilization in children with CP. Ankle joint mobilization can reduce COP displacement by improving sensorimotor function and arthrokinematic restrictions [25]. For the elderly, ankle joint mobilization reduces the surface of standing COP excursions [49]. However, children with CP maintain postural control using a body sway rather than ankle strategy in a quiet standing position [8]. Because of poor postural control, children with CP typically have increased static COP displacement compared to developing children [16]. The wearing of hinged ankle–foot orthoses increased ankle strategy contribution but did not improve postural stability in quiet standing [10]. Therefore, it seems that there was no change in standing balance due to the contribution of body transverse rotation and hip strategies [8,16]. In this study, an NDT program consisted of improving trunk control. An NDT-based trunk protocol is beneficial in improving the trunk control and balance in children with spastic diplegic CP [37]. Therefore, improvement of trunk control by an NDT program decreased COP displacements. Since both groups performed the NDT program, the improvement of trunk control and balance by the NDT program may cause no significant difference between the two groups in COP displacements.

Additionally, the COP displacements in this study evaluated the total COP trajectory. The wearing of hinged ankle–foot orthoses did not change the anterior and posterior displacements but increased the mediolateral displacements [16]. Therefore, to confirm the COP displacements for ankle dorsiflexion ROM increase, it is necessary for future studies to check the mediolateral displacements and anterior–posterior displacements changes, respectively.

Manual therapy applied to growing children can stimulate skeletal growth [30]. Fortunately, no serious or catastrophic adverse events have been reported for children [50,51]. However, due to insufficient evidence so far with regard to manual therapy and adverse events, caution should still be exercised [30,50].

Spine manipulation reduced wrist muscle spasticity in children with CP [27]. Ankle joint mobilization also reduced ankle muscle spasticity in brain injury or incomplete spinal cord injury patients [33]. In this study, ankle joint mobilization, which is safer than spine manipulation, was applied. Joint mobilization can stop the treatments on its own whenever the patient wants to [28]. Therefore, we hope that joint mobilization is often used in clinical settings because ankle joint mobilization is a safe treatment method.

The limitations of this study are as follows. First, our study has a small sample size and it is thus difficult to generalize, and also because our recruitment was limited to GMFCS level I or II. Secondly, our walking speed evaluation method is an evaluation frequently used in clinical settings, but ankle kinematic changes during gait and spatiotemporal analysis (motion capture or inertial measurement units) were not confirmed. To support our hypothesis, we need to further refine and systematically evaluate gait analysis. Finally, this study did not compare long-term effects. Complementing these limitations, future research should investigate the impact of a greater sample size, multiple assessments, and long-term follow-up.

## 5. Conclusions

The present study shows that additional ankle joint mobilization improves ankle ROM and gait in children with CP. However, the beneficial effect on standing balance was not confirmed. The present study provides new clinical evidence of ankle joint mobilization to increase ankle movements in children with CP. Future research should investigate the impact of a greater sample size, multiple assessments, and long-term follow-up studies.

## Figures and Tables

**Table 1:** General characteristics of the recruited subjects.

Classification	Experimental Group (n = 16)	Control Group (n = 16)	*p*-Value ^b^	*p*-Value ^c^
Gender (male/female)	9/7	10/6	1.000	
GMFCS (Level I/Level II) ^d^	6/10	6/10	1.000	
Age (years) ^a^	10.81 ± 2.34	11.31 ± 1.78		0.502
Weight (kg) ^a^	37.88 ± 8.17	37.13 ± 6.64		0.778
Height (cm) ^a^	137.31 ± 9.67	139.06 ± 7.77		0.577

^a^ Values are denoted as the mean ± SD. ^b^ Chi-square test among two intervention groups. ^c^ Independent *t*-test among two intervention groups. ^d^ GMFCS: Gross motor function classification system.

**Table 2:** Comparison in dorsiflexion ROM pre- and post-test.

Measure/Group	Pre-Test ^a^	Post-Test ^a^	Within-Group Difference ^b^	Between-Group Difference ^b^
Lt. Ankle dorsiflexion ROM in sitting position (°)				
Experimental group	14.31 ± 3.00	18.00 ± 2.16	3.69 (2.70, 4.67) *	3.44 (2.42, 4.46) **
Control group	13.63 ± 2.45	13.88 ± 2.83	0.25 (−0.06, 0.56)	
Rt. Ankle dorsiflexion ROM in sitting position (°)				
Experimental group	14.38 ± 3.16	18.56 ± 2.06	4.19 (2.98, 5.39) *	3.94 (2.71, 5.17) **
Control group	13.69 ± 2.55	14.06 ± 2.77	0.37 (−0.01, 0.76)	
Lt. Ankle dorsiflexion ROM in supine position (°)				
Experimental group	8.88 ± 2.53	11.56 ± 1.93	2.69 (1.89, 3.48) *	2.31 (1.43, 3.20) **
Control group	7.69 ± 2.50	8.06 ± 2.54	0.38 (−0.10, 0.85)	
Lt. Ankle dorsiflexion ROM in supine position (°)				
Experimental group	9.00 ± 2.83	12.44 ± 2.50	3.44 (2.50, 4.37) *	2.94 (1.87, 4.01) **
Control group	7.81 ± 2.59	8.31 ± 2.36	0.50 (−0.12, 1.12)	

^a^ Values are the means ± SD. ^b^ Values are the 95% confidence intervals. * Within-group factors: Significant increase compared to the pre-test. ** Between-group factors (Interaction): Significant increase compared to the control group. Experimental group: Ankle joint mobilization. Control group: Sham joint mobilization. ROM: Range of motion.

**Table 3:** Comparison of COP displacements, TUG, and 10-m walk test pre and post-test.

Measure/Group	Pre-Test ^a^	Post-Test ^a^	Within-Group Difference ^b^	Between-Group Difference ^b^
Static sway length in the eyes opened condition (cm)				
Experimental group	12.74 ± 2.94	10.23 ± 2.41	−2.50(−3.32, −1.68) *	−0.79 (−1.85, 0.25)
Control group	12.78 ± 2.77	11.07 ± 2.50	−1.71(−2.43, −1.86) *	
Static sway area in the eyes opened condition (mm^2^)				
Experimental group	53.35 ± 6.85	36.66 ± 8.73	−16.69(−21.50, −11.87) *	−2.93 (−9.20, 3.34)
Control group	54.15 ± 7.10	40.39 ± 7.58	−13.76(−18.19, −9.33) *	
Static sway length in the eyes closed condition (cm)				
Experimental group	14.28 ± 2.41	11.23 ± 2.37	−3.05(−4.03, −2.07) *	−0.98(−2.06, 0.09)
Control group	14.42 ± 2.30	12.36 ± 2.61	−2.06(−2.61, −1.51) *	
Static sway area in the eyes closed condition (mm^2^)				
Experimental group	59.11 ± 9.14	39.43 ± 9.98	−19.67(−23.99, −15.35) *	−1.34(−11.57, 2.89)
Control group	59.83 ± 9.10	44.49 ± 9.84	−15.34(−21.52, −9.15) *	
TUG test (sec)				
Experimental group	12.24 ± 2.06	8.50 ± 2.76	−3.74(−4.45, −3.02) *	−1.95(−3.12, −0.78) **
Control group	12.78 ± 2.09	11.00 ± 1.68	−1.78(−2.77, −0.79) *	
10 MWT (sec)				
Experimental group	10.02 ± 2.26	7.61 ± 1.96	−2.41(−2.95, −1.86) *	−1.39(−2.10, −0.68) **
Control group	11.09 ± 1.98	10.08 ± 1.75	−1.01(−1.51, −0.51) *	

^a^ Values are the means ± SD. ^b^ Values are the 95% confidence intervals. * Within-group factors: Significant decrease than the pre-test. * Within-group factors: Significant decrease compared to the pre-test. ** Between-group factors (Interaction): Significant decrease compared to the control group. Experimental group: Ankle joint mobilization. Control group: Sham joint mobilization. TUG test: Timed up and go test. 10 MWT: 10-m walk test.
